# Drought, Wellbeing and Adaptive Capacity: Why Do Some People Stay Well?

**DOI:** 10.3390/ijerph17197214

**Published:** 2020-10-02

**Authors:** Emma K. Austin, Tonelle Handley, Anthony S. Kiem, Jane L. Rich, David Perkins, Brian Kelly

**Affiliations:** 1 Centre for Water, Climate and Land (CWCL), Faculty of Science, University of Newcastle, Callaghan 2308, Australia; Anthony.Kiem@newcastle.edu.au; 2 Centre for Rural and Remote Mental Health (CRRMH), Faculty of Health and Medicine, University of Newcastle, Orange 2800, Australia; Tonelle.Handley@newcastle.edu.au (T.H.); David.Perkins@newcastle.edu.au (D.P.); 3 Centre for Brain and Mental Health Research (CBMHR), Faculty of Health and Medicine, University of Newcastle, Callaghan 2308, Australia; Jane.Rich@newcastle.edu.au; 4 School of Medicine and Public Health, Faculty of Health and Medicine, University of Newcastle, Callaghan 2308, Australia; Brian.Kelly@newcastle.edu.au

**Keywords:** drought, wellbeing, adaptive capacity, salutogenesis, sense of coherence

## Abstract

Drought is a threat to public health. Individual and community adaptive capacity is crucial when responding to the impacts of drought. Gaps remain in the understandings of the relationship between wellbeing and adaptive capacity, and whether increased wellbeing can lead to improved adaptive capacity (or vice versa). This paper explores the relationship between drought, wellbeing and adaptive capacity to provide insights that will inform actions to enhance adaptive capacity, and hence increase opportunities for effective drought adaptation. The theory of salutogenesis and the associated sense of coherence (SOC) are used to measure adaptive capacity and to explain why some individuals remain well and adapt to adversity while others do not. An online survey of rural residents (*n* = 163) in drought-affected New South Wales (NSW), Australia, was conducted from November 2018 to January 2019. Linear regression was used to model the relationships between SOC, sociodemographic factors, drought and wellbeing. Findings demonstrate that SOC is strongly correlated with wellbeing. Drought condition did not influence adaptive capacity, although adaptive capacity and drought-related stress were only weakly correlated. Increased wellbeing was found to be associated with stronger adaptive capacity and therefore, an individuals’ capacity to cope with adversity, such as drought.

## 1. Introduction

Individual and community adaptive capacity is essential when responding to the impacts of drought [1]. Increased understanding of the relationship between adaptive capacity and wellbeing is needed to provide insights into methods to enhance adaptive capacity, and therefore increase opportunities for effective drought adaptation. Many definitions of adaptive capacity, and its interconnections with vulnerability and resilience, are available [2,3,4,5,6]. In addition, there are numerous methods and techniques for assessing adaptive capacity, such as assessment of secondary data sources, inductive theory-driven approaches, futures modelling and self-assessment processes [7]. Despite these methods and techniques, an optimal measure of adaptive capacity has not been identified. For this reason, it is necessary to assess alternative methods for measuring adaptive capacity.

This paper uses Antonovsky’s theory of salutogenesis and the associated sense of coherence (SOC) [8] to measure adaptive capacity in rural residents affected by drought. The concept of salutogenesis posits that SOC is a method of understanding why some individuals cope and adapt to adversity and remain healthy, while others do not [9,10,11,12]. In this case, adversity is represented by drought. Antonovsky theorised that a person’s perspective on their life has an influence on their health, where a positive view of life results in a positive influence to health [8,13]. Specifically, the SOC explains why some people stay well in stressful situations and is helpful in managing daily stressors and life events. Research shows that many people in drought-affected communities do indeed cope and adapt to drought successfully while remaining well, highlighting the relevance of SOC to this study, as a way of understanding why and how this is possible under the ongoing stress of drought.

In this paper, adaptive capacity is conceptualised as a nested concept within the context of resilience and vulnerability [6,14], symbolising an individual’s ability to cope with adversity, including drought. Vulnerability, and resilience to drought differs across locations and populations, with sociodemographics, health and financial position contributing to an individual’s adaptive capacity and ability to cope and adapt to drought [15,16].

### Salutogenesis and the Sense of Coherence (SOC)

Understandings of salutogenesis, and the associated SOC, are employed as a concept of health and as a proxy for adaptive capacity. The concept of salutogenesis was conceived by Aaron Antonovsky, a medical sociologist studying the health of women in Israel, including some who were concentration camp survivors [17]. The concept originated from Antonovsky’s discovery that some women who had survived the horrors of a concentration camp, and then been displaced, had better emotional and physical health than women who had not been imprisoned.

Theories of health are typically disease focused, however, salutogenesis offers an alternative view, instead guided by the question “what makes people healthy?” with a focus on health and health assets, rather than the origins of disease and risk factors [18]. The theory originated from Antonovsky’s insight that illness, however it manifests, was a consequence of psychosocial factors [18]. Antonovsky hypothesised that our life experiences shape our SOC, defined as: “A global orientation that expresses the extent to which one has a pervasive, enduring though dynamic feeling of confidence that one’s internal and external environments are predictable and that there is a high probability that things will work out as well as can be expected.” [8] (p. 123)

An individual’s SOC strength is considered as a crucial element in the structure of their personality, facilitating the coping and adaptation process [13]. SOC is the mechanism that facilitates us to employ our resources to cope with stressors. Understanding these stressors and the role they may play in health and wellbeing is important when contextualising and defining human health. SOC also provides a fresh way of examining health and wellbeing in the science/public health disciplines. Used to conceptualise and measure adaptive capacity in this paper, SOC acknowledges the adversities people face and their ability to experience resilience.

SOC has been found to impact quality of life: where stronger SOC results in better quality of life [19]. Antonovsky [8] described SOC as the ability to comprehend the whole situation and the associated capacity to use the resources available to cope with the situation. This description determines that SOC has three elements [20]:

- Comprehensibility—the cognitive dimension—refers to the level at which one perceives internal and external stimuli as rationally understandable. This understanding is critical, as being able to cope with a stressful situation is dependent on one’s ability to understand it to some extent, where comprehension makes it easier to manage.
- Manageability—the instrumental or behavioural dimension—is the extent to which one feels confident there are the resources available to meet the requirements of the stimuli. Critically, coping also requires one to be (i) motivated to solve the problems causing the stressful situation, while being (ii) willing to invest energy in solving the problems, and (iii) finding meaning in being able to manage the situation. This leads to the third element.
- Meaningfulness—the motivational dimension—refers to the degree to which one feels life has an emotional meaning. Essentially, one must feel that the problems faced in life are worth commitment and dedication, viewing these problems as challenges instead of burdens. Finally, one must have a desire to resolve problems, and a willingness to invest energy to survive stressful experiences.

Antonovsky [8] theorised that an individual’s SOC was determined by their general resistance resources (GRRs). There are six categories of GRRs: physical, artefactual, emotional, cognitive, macrosocial and social [10]. Despite these definitions, SOC is not a coping strategy per se, rather a high SOC increases the likelihood that an individual will flexibly adopt adaptive strategies which are appropriate to the given situation [9]. Ultimately SOC relates to the resources, mechanisms and interactions that guide and facilitate the adaptive capacity of humans [10].

Although, SOC has been used in a variety of methodologies, including anonymous random surveying [9] and qualitative interviews [10], there exist no studies where SOC was explicitly used to measure adaptive capacity. Therefore, the research presented in this paper uses SOC to measure the adaptive capacity of rural residents in order to assess its usefulness in further exploring these issues. In addition, while salutogenesis and SOC have been used in a multitude of scenarios and contexts in more than 49 languages in at least 48 countries [11] they have not been applied in a drought situation, indeed they have not been utilised in the context of environmental or climatic impacts to health.

Applying the theory of salutogenesis and SOC in this way provides an opportunity to learn from experimenting with varied health theories when considering the relationship between drought and wellbeing. Salutogenesis and SOC have not been applied in this context previously, allowing this new application to provide novel insights. While numerous theories and measures of resilience and adaptive capacity exist, salutogenesis was chosen as the guiding theory as it incorporates elements of resilience and adaptive capacity together with health and wellbeing specifically. Importantly, SOC helps to delineate why some individuals thrive despite adversity while others do not. Establishing the linkages between this ability to thrive and wellbeing is central to the aim of this paper, as an understanding of this relationship may contribute to improving adaptive capacity to drought.

In this paper, wellbeing was measured using the K10, in which the absence of psychological distress represents positive wellbeing. While K10 (i.e., level of psychological distress) was deemed to be a sufficient measure of wellbeing for the purpose of this paper it is important to recognise that wellbeing also encompasses many other parameters (e.g., physical health, financial position, satisfaction with relationships etc.).

## 2. Materials and Methods

### 2.1. Study Area and Population

**Figure 1:**
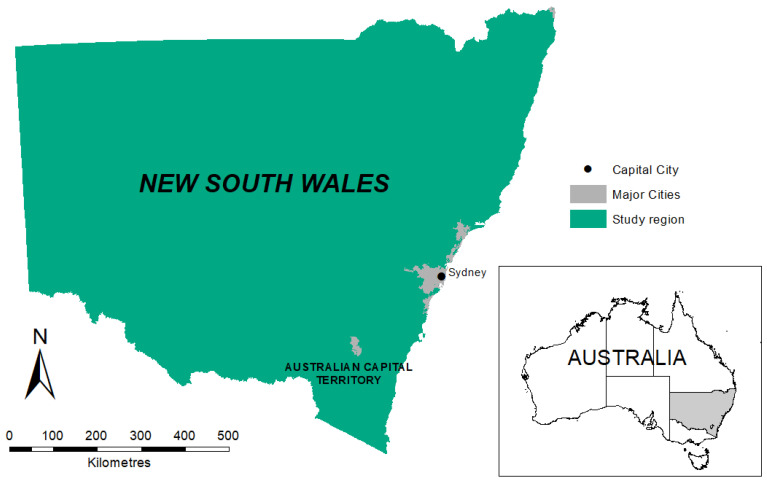
Study region—non-metropolitan New South Wales (NSW), Australia.

The study region for this research was non-metropolitan New South Wales, Australia (Figure 1). Data were gathered via an online survey conducted from November 2018 to January 2019 using Research Electronic Data Capture (REDCap) [21] to determine associations between adaptive capacity and: (i) sociodemographic and community factors; (ii) drought; and (iii) wellbeing. To be eligible participants needed to be 18 years or over and reside in New South Wales (NSW) outside a major city. Eligibility was determined by two initial screening questions. The survey included open and closed questions, providing participants with the opportunity to detail methods of climate adaptation they had used in the past, were currently using or intended to use in the future. Recruitment was carried out via social media and websites (e.g., the Centre for Rural and Remote Mental Health [22], Rural Adversity Mental Health Program (RAMPH) [23] and the University of Newcastle [24], radio interviews, and snowballing via emails to networks and colleagues. All participants who completed the survey to the end were eligible to go into a draw to win a gift card incentive worth AUD 400. The survey was approved by the human research ethics committee at the University of Newcastle (approval number H-2018-0373).

### 2.2. Outcome Measure

SOC is measured by the sense of coherence scale, originally the orientation to life questionnaire [13]. In this paper, the 13-item version of the SOC (SOC13) is used. Permission to use the SOC13 was granted by the head of the centre on salutogenesis, department of health sciences, university west, Sweden.

The SOC13 is a seven-point semantic differential scale; designed to measure connotative meaning, where the connotations are then used to derive the attitudes towards the object, event or concept under investigation [25]. Using this measuring tool requires respondents to select their position on a scale between two polarised adjectives (e.g., “good” or “bad”) [26]. Examples of questions in the SOC13 include: “Has it happened in the past that you were surprised by the behaviour of people whom you thought you knew well?” and “Has it happened that people whom you counted on disappointed you?” These questions were answered on a range from “never happened” to “always happened.”

Total SOC13 scores range between 13 and 91. Total and average SOC were calculated from the SOC13 responses. Total SOC was categorised as weak (13–63) and strong (64–91) [27], whereby strong SOC represents the capacity to adapt, cope, and remain well. As intended, the SOC13 items 1–3, 7 and 10 were reverse-scored due to negative wording (i.e., the polar opposites of response options were reversed due to the negative wording of the questions) [13].

### 2.3. Influencing Factors

The influencing sociodemographic and community factors identified in Austin et al. [28] as being significant for drought-related stress were tested for associations with the SOC13. Wellbeing was measured with the K10. Total personal (PDS) and community (CDS) drought-related stress were also considered as influencing factors. Due to the lack of power because of the small sample size, testing additional factors was not possible.

The drought index of months below precipitation decile 1 (i.e., very much below average) were calculated at the 24-month time window, as per findings from Austin et al. [29] which concluded that this index and time frame were optimal for capturing K10 across the four postcodes used as case studies in that analysis. Rainfall data used were from the Australian water availability project (AWAP), which monitors the state and trend of the terrestrial water balance of the Australian continent, using model-data fusion methods to combine measurements and model predictions [30,31].

### 2.4. Analysis

Data were analysed using SPSS (version 24; Armonk, NY, USA). Univariate analysis was performed for SOC13, sociodemographic factors, drought condition, drought-related stress and K10. Categorical variables were tested using ANOVA and continuous variables were tested with Pearson’s correlation coefficient. The categories for Pearson’s coefficient are the same for positive and negative (i.e., 1.0 to −1.0) (Table 1). Linear regression was used to model the relationship between SOC13 and: (i) drought-related stress and the sociodemographic and community factors that were identified as significant for drought-related stress in Austin et al. [28]; (ii) drought condition (as determined in Austin et al. [29]); and (iii) wellbeing.

## 3. Results

### 3.1. Population

**Figure 2:**
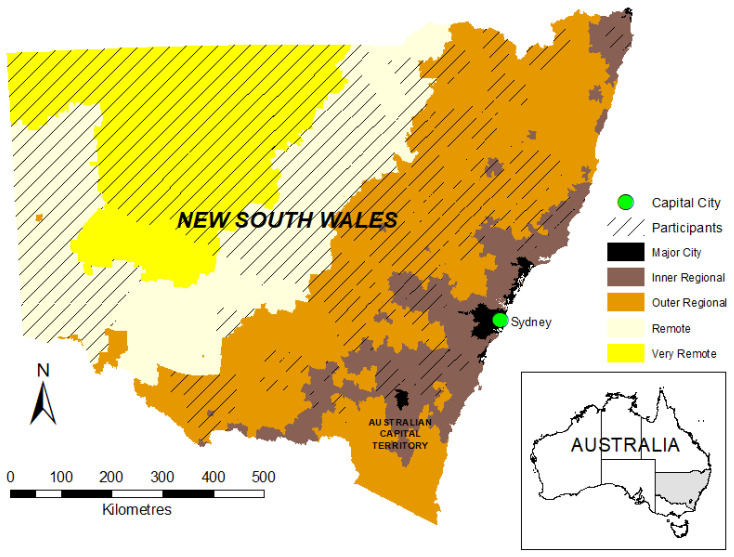
Location of study population in New South Wales (NSW) according to postcode and remoteness class [33].

The survey was commenced by 221 participants; 20 were either deemed ineligible by the initial screening questions relating to location and age or exited after giving consent without completing any responses. Only those that answered all items in both the K10 and SOC13 were included, giving a final population of 163. The location of participants is shown in Figure 2. It is acknowledged that an Aboriginal and Torres Strait Islander perspective of drought, wellbeing and adaptive capacity is not captured in this research, as the survey did not ask participants whether they identified as Indigenous. Targeting Aboriginal and Torres Strait Islanders specifically was outside the ethics approval granted for this project.

The sociodemographic characteristics of the study population are provided in Table 2. In summary, 72.4% of participants were women, more than half (52.1%) of the population were aged 45–64 years, and the majority of participants lived and worked on a farm (54.0%). Almost half the population (47.9%) reported their financial position as just getting along, 41.7% had a university degree or higher, and most participants were married (either officially or de facto) (80.3%). The most common occupations were: farmers and farm managers (33.1%), professionals (18.4%) and community and personal service workers (8.0%). Of the farmers and farm managers, the main commodities were livestock (22.7%) and mixed crop and livestock (9.8%).

When asked how often they experienced worries or stress about drought, participants’ responses from the Likert scale were: always (22.1%), very frequently (46.6%), occasionally (24.5%) and rarely (6.7%). No participants reported “never” experiencing drought worry.

**Figure 3:**
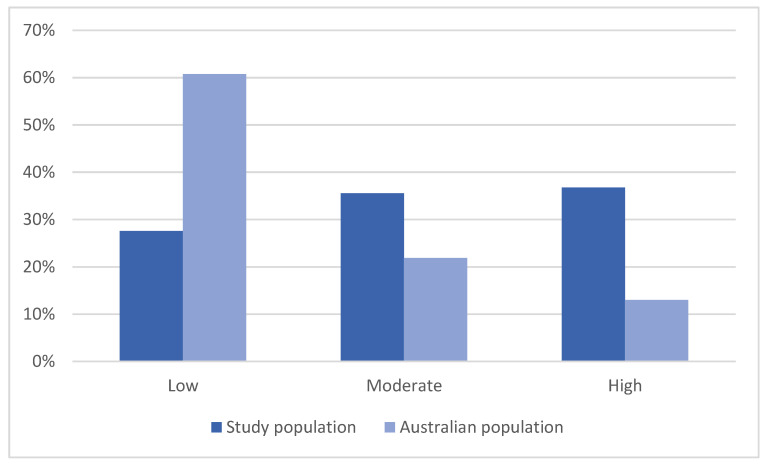
Comparison of levels of psychological distress between the study and Australian populations [34].

The average K10 for the study population was 21.89. High distress was reported by 36.8% of the study population, compared to 13.0% of the Australian population in general (Figure 3) [34]. Comparison of average K10 with previous studies in non-metropolitan NSW among the general rural and remote population demonstrates the increased level of distress of the population in this study: Austin et al. [35] average K10 = 13.83, Austin et al. [28] average K10 = 14.24, and Austin et al. [29] average K10 = 14.31.

### 3.2. Drought-Related Stress

Individual items of PDS and CDS are shown in Table 3. Compared to the populations in previous research, drought-related stress for all individual items was higher than for the population in Austin et al. [28], and similar to Austin et al. [29], reinforcing that this population was particularly distressed. Except for loss of contact with friends (an item of PDS) and people leaving the area (an item of CDS) all other individual items of both PDS and CDS were experienced by more than 50% of the study population.

### 3.3. Sense of Coherence

The SOC13 was designed to be interpreted as a summed score; the individual items were not intended to be considered separately. Scores ranged from 25 to 88 with a mean score for the whole population of 61.6 (SD 14.5) which is categorised as a weak SOC. This mean SOC is low when compared to other populations studied. For example, mean SOC was 70.7 for healthy 40–70 year olds in Finland [36] while an investigation of nurses in Greece returned a mean SOC of 63.6 [37].

Univariate analysis of sociodemographic variables found financial position to be the only significant factor associated with SOC strength (χ2(8) = 18.9; *p* < 0.001), where those reporting financial hardship also reported weaker SOC. Although not statistically significant, women were more likely to have weak SOC while more men had strong SOC. This gender pattern has been found previously [37,38]. Younger participants (18–54) more often had weak SOC, with SOC strength increasing with age, which is consistent with previous findings [20]. Participants who lived and worked on a farm reported weaker SOC compared to other participants.

### 3.4. Current Climate Adaptation

The majority (82.2%) of participants reported that they were currently engaging in climate adaptation. Participants’ current climate adaptation practices are shown in Table 4. When tested using chi-square, current climate adaptation category was not associated with SOC13 (χ2(5) = 0.02, *p* = 0.888) or K10 (χ2(9) = 0.984, *p* = 0.611); therefore SOC and wellbeing were not related to current adaptation. Due to the sample size, it was not feasible to test the relationship with only those participants who identified as farmers. It is possible the results would have differed if the relationship were tested only with farmers.

**Figure 4:**
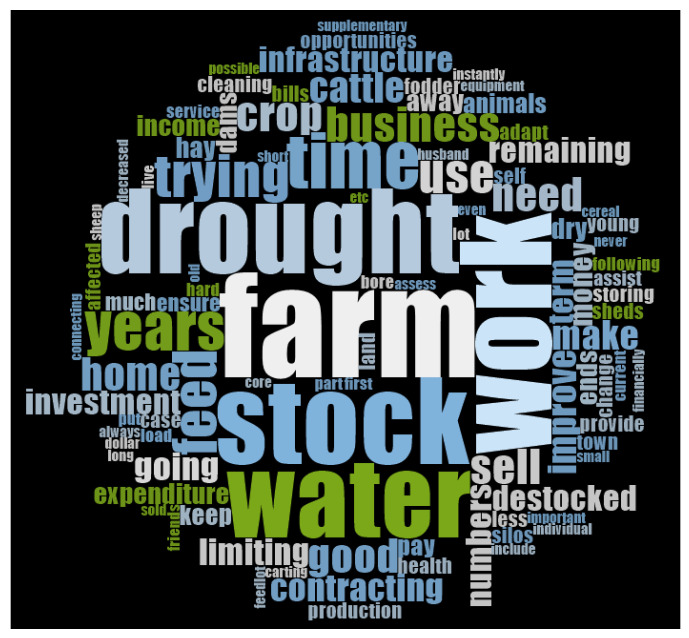
Word cloud showing the most common words used by participants in free-text responses when asked about the methods they were currently using to adapt to drought.

The survey provided participants with the opportunity to give further details about their current adaptation practices to gain insights into the relationship between adaptation and wellbeing. The most frequently used words in these free-text responses are shown in Figure 4. The figure is a word cloud generated in NVivo. The size of the words in the word cloud represents how many times the words were used in responses, relative to other words [39]. Although the method is similar to a word frequency table, word clouds have the benefit of allowing visual assessment of the results in an attractive and easily interpreted manner [40]. The use of this assessment method is increasing in a number of disciplines [41]. From this visualisation, it is apparent that adaptation practices were related to farming and drought and focused on issues associated with stock, water and work. Future research should employ content analysis and/or thematic analysis to analyse these qualitative data further. Conclusions drawn from the method used here should be interpreted with caution.

### 3.5. Factors Associated with SOC13

**Figure 5:**
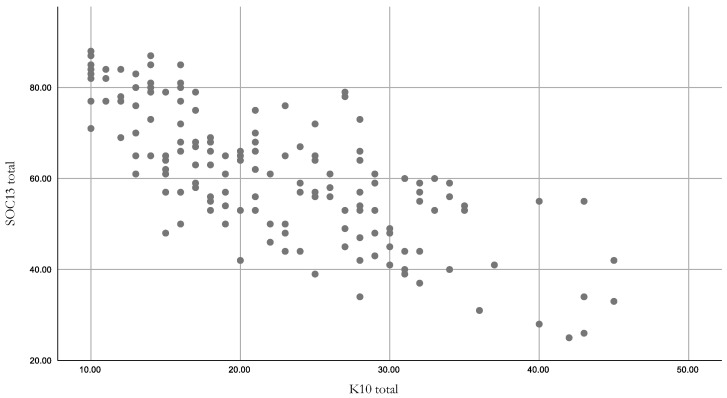
Scatter plot showing correlation between SOC13 and K10 (N.B. SOC13 strength: weak 13–63; and strong 64–91).

Univariate analysis of the summed totals of SOC13 and K10 showed they were strongly (Table 1) negatively correlated with each other (*r* = −0.76; *p*-value < 0.001) (Table 5 and Figure 5). This is consistent with other investigations into the relationship between SOC and psychological distress variables [42]. There was a stronger correlation at the lower end of the SOC13 (i.e., when SOC is weak and psychological distress is high). Individual items of the SOC13 and K10 were associated with weaker correlations. There were weak negative correlations between SOC13 and drought-related stress (PDS *r* = −0.39, *p*-value < 0.001; CDS *r* = −0.37, *p*-value < 0.001). Similar to K10, SOC was weaker when drought-related stress was higher. Similar to findings in Austin et al. [28], K10 had weaker correlations with PDS (*r* = 0.39, *p*-value < 0.001) and CDS (*r* = 0.39, *p*-value < 0.001).

When SOC13 was correlated with the categorical sociodemographic factors, age and financial position had statistically significant differences between groups (Table 6). SOC was higher among older age groups, which is consistent with findings by Eriksson [20] where participants who were 55+ years had a mean SOC13 score in the strong range (64–91), while younger participants’ mean SOC13 was in the weak category (13–63). Financially secure participants also had a mean SOC13 score in the strong SOC range, with participants experiencing financial hardship also reporting weak SOC. This relationship was consistent for K10; participants experiencing financial hardship reported higher psychological distress.

Linear regression was used to further investigate the relationship between SOC and influencing factors. The models were grouped as: Model I sociodemographics and drought-related stress; Model II drought condition; and Model III wellbeing. Financial position was statistically significant for Models I (β = 0.41; *p* < 0.001) and II (β = 0.40; *p* < 0.001), with financially secure participants reporting a stronger SOC (Table 7). Personal drought-related stress was associated with a weaker SOC in Models I (β = −0.24; *p* < 0.05) and II (β = 0.02; *p* < 0.05). Financial position and drought-related stress were not significant in Model III when K10 was added, with K10 associated with weaker SOC (β = −0.71; *p* < 0.001) as initially demonstrated in the univariate analysis.

### 3.6. Drought Condition

**Figure 6:**
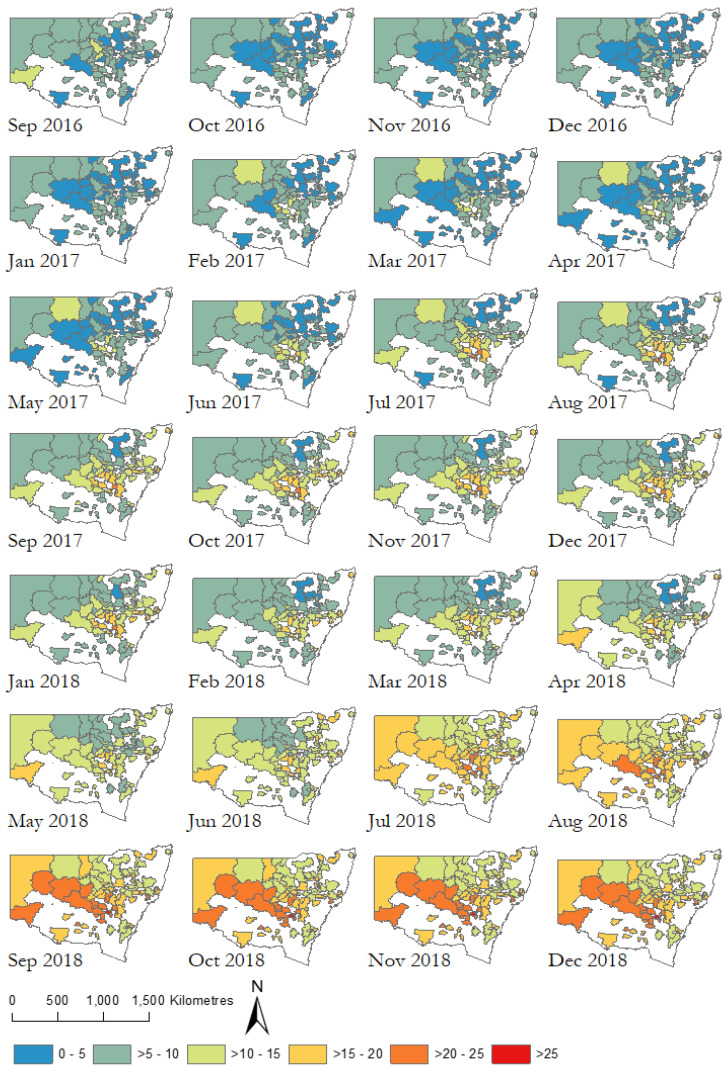
Percentage of months below precipitation decile 1 (24-month time window) for the postcodes where participants resided.

During the 24 months prior to the survey commencing, there was a gradual spread of drought conditions across NSW (Figure 6). Drought condition is measured for the month prior to the survey completion (e.g., survey responses in January 2019 use the drought condition in December 2018). During the six months leading up to the survey, all participants had experienced a minimum of 10–15% of months in drought in the past 24 months, and a maximum of 25% of months in drought. This highlights the extent of drought conditions experienced by the study population. In addition, the drought condition for many participants had been escalating during the time of data collection for this study (November 2018–February 2019). It is important to note that the K10 asks about feelings in the past four weeks. This timing is important as it corresponds with the intensification and culmination of drought conditions. It is necessary to consider the timing of the survey compared to the propagation of drought conditions and the period of the K10. Although distress was high in the group overall, there was no association between distress and the amount of time spent in drought.

Average SOC for each level of distress according to drought condition is shown in Table 8. Participants with low distress reported stronger average SOC regardless of drought condition while participants with high distress reported weaker average SOC. The highest number of months in drought (>25%) had the weakest average SOC (57).

## 4. Discussion

Adaptive capacity, measured by the SOC13, was found to be strongly correlated with wellbeing as measured by the K10, a finding supported by previous investigations of the relationship between SOC and psychological distress variables [37,42]. Drought condition did not influence adaptive capacity, although adaptive capacity and drought-related stress were weakly correlated. Adaptive capacity improved with age and financial security, consistent with the findings for drought-related stress in Austin et al. [28]. Psychological distress and drought-related stress were higher than in other populations investigated previously [28,35], and importantly no participants reported never being worried about drought. These findings demonstrate that adaptive capacity and wellbeing are linked and that improved wellbeing in terms of lower psychological distress should enhance adaptive capacity.

These findings highlight the importance of baseline data, and being able to compare populations before/after they are disturbed by any extreme event. Such a large percentage of the study population reporting high distress (i.e., nearly triple that of the general Australian population) highlights the importance of funding and programs to support people in rural communities affected by drought, regardless of whether this high distress is caused directly by drought. Research such as that described here and previously, e.g., [16,28,35,43], help to identify populations most at risk of diminished wellbeing as a result of drought. Participants with high distress were more likely to have weaker SOC, suggesting that increased wellbeing can help people remain well and be able to cope with stressors and adversity, including drought.

Reporter bias, the use of snowball sampling and the inability to calculate a response rate are limitations of this study. It is likely that only those affected by drought and potentially only those who were distressed, responded to the survey. This is a factor to consider when recruiting and framing the advertising material associated with surveys such as this. In addition, the use of an online survey may introduce bias, as people with no access to the internet, or who are not comfortable using it, may not have responded. Finally, the population may be a survivor cohort, in that those with low adaptive capacity have already exited the drought-affected area.

Compromised wellbeing, specifically anxiety and depression, are directly linked to how individuals perceive the likelihood of future events [44]. Anxiety and depression generate more negative future thinking and are associated with elevated levels of worry and hopelessness [44]. Strong SOC helps individuals to manage the lack of control over their life and feelings of instability. In this way, SOC facilitates the coping and adaptation process. This is important in the context of drought, as drought propagates over time and as conditions deteriorate people’s wellbeing is influenced by the prospect of future rain. Indeed, Ellis and Albrecht [45] reported some farmers checking the forecast up to 20 times a day in the hope for rain. This manifests into a type of social sorrow, where hopes for the future hinge on rainfall, and people are subjected to repeated disappointment when it does not rain.

Findings demonstrate that wellbeing and adaptive capacity was at its lowest for 10–15% of months in drought (in a 24-month period), which suggests that as drought continued wellbeing and adaptive capacity improved. From analyses conducted here it is not possible to fully interpret why distress may be higher in times of lesser drought. One possible scenario is that people are more distressed as drought develops, and once the drought reaches a tipping point, distress is reduced. This situation may be explained by the possibility that people have started to adapt (e.g., changes to household budget, farming practices or lifestyle) to drought or that government funding has become available. Further analysis, including qualitative investigations, are needed to fully explain this relationship.

Austin et al. [28] and Austin et al. [29] found a significant relationship between drought condition and wellbeing, while Austin et al. [35] qualitatively reported on this linkage. Despite these previous findings, a relationship was not detected between drought condition and adaptive capacity in this study, although greater drought-related stress was associated with lower adaptive capacity. However, the population in this study is very different to those in the previously published papers. The population in this paper differs in three important ways: (i) it is a much smaller sample size; (ii) it is almost exclusively drought-affected; and (iii) is a considerably distressed sample (although it is not possible to determine the cause/s of this heightened distress). These methodological limitations may have impacted the reliability of the findings. Research demonstrates that wellbeing in rural communities is not wholly controlled by drought experience, and a range of factors influence wellbeing and drought-related stress [1,28,46]. This is supported by findings in this paper, as although drought was not found to have a statistically significant effect, the prevailing drought conditions cannot be ignored when interpreting the high levels of distress.

## 5. Conclusions

Despite the literature suggesting a relationship between SOC and adaptive capacity, SOC has not previously been used to measure adaptive capacity. While adaptive capacity was not associated with drought condition, it was found that increased wellbeing is linked to improved adaptive capacity. These findings highlight the importance of having baseline data for rural communities that are vulnerable to drought so that comparisons are possible when a drought (or another extreme event) occurs. While SOC has been applied to a variety of contexts and methods, this study is the first to test SOC as a measure of adaptive capacity, as well as being the first time SOC has been used in an environmental adversity context.

It is necessary to compare and identify influencing factors so that support can be targeted to those most at risk to maximise the efficacy of funding and community interventions. Adaptation to drought is essential as drought is a reoccurring pervasive element of our climate. Findings reported here suggest increased wellbeing is associated with stronger adaptive capacity and therefore, an individuals’ capacity to remain well and cope with stressors, such as drought and rural adversity. It remains to be investigated if interventions to promote comprehensibility, manageability and meaningfulness in drought-affected populations can improve adaptive capacity to drought.

## Figures and Tables

**Table 1:** Example of interpretations of the correlation coefficient [32].

Correlation Coefficient		Interpretation of Correlation
0.00 to 0.10	0.00 to −0.10	Negligible
0.10 to 0.39	−0.10 to −0.39	Weak
0.40 to 0.69	−0.40 to −0.69	Moderate
0.70 to 0.89	−0.70 to −0.89	Strong
0.90 to 1.00	−0.90 to −1.00	Very strong

**Table 2:** Sociodemographic characteristics of the study participants.

Characteristics	*n*	%	Characteristics	*n*	%
Farmer Status			Financial Hardship		
Live and work on a farm	88	54.0	Very comfortable	13	8.0
Live on a farm	28	17.2	Reasonably comfortable	57	35.0
Work on a farm	6	3.7	Just getting along	78	47.9
Rural resident and neither work nor live on a farm	35	21.5	Poor	11	6.7
			Very poor	4	2.5
None of the above	6	3.7	Completed school		
Gender			No school or other qualification	2	1.2
Female	118	72.4	School certificate or equivalent	16	9.8
Male	44	27.0	Higher school certificate or equivalent	28	17.2
Other	1	0.6	Trade/apprenticeship	8	4.9
Age			Certificate/diploma	41	25.2
18–34	25	15.3	University or higher degree	68	41.7
35–44	34	20.9	Remoteness (ASGC*)		
45–54	40	24.5	Inner regional	10	6.1
55–64	45	27.6	Outer regional	91	55.8
65+	19	11.7	Remote	24	14.7
Lived in current postcode			Very remote	38	23.3
1 year or less	5	3.1	Marital status		
1–2 years	9	5.5	Married/De facto	131	80.3
3–5 years	12	7.4	Separated/Divorced	9	5.6
6–10 years	21	12.9	Widow	2	1.2
More than 10 years	73	44.8	Never married	21	12.9
Whole life	43	26.4			
Employment status					
Employed/Home duties/Studying	147	90.1			
Unemployed/Unable to work	7	4.3			
Retired	8	4.9			

* ASGC—Australian Standard Geographical Classification.

**Table 3:** Proportions of participants who experienced individual items of personal (PDS) or community (CDS) drought-related stress.

	Individual Items	*n*	%
PDS	Money/financial pressures	121	74.2
	Business pressures	96	58.9
	Loss of contact with friends	71	43.6
	Not going out as much	97	59.5
	More work to do	125	76.7
	Less time for family	100	61.3
CDS	People leaving the area	72	44.2
	Losing business and services in town	96	58.9
	Not getting together as much	96	58.9
	Countryside has changed	144	88.3
	Reduced water quality	101	62.0

**Table 4:** Current climate adaptation practices.

Adaptation Method	*n*	%
Not currently adapting	29	17.8
Farming practices	107	65.6
Business structure	63	38.7
Off-farm employment	64	39.3
Crop diversification or change	41	25.2
Other (provided free-text response)	63	38.7

**Table 5:** Univariate analysis to test correlations between the 13-item version of the sense of coherence (SOC) scale (SOC13), K10, PDS, CDS and drought condition.

		SOC13	PDS	CDS	Drought	K10
SOC13	*r*		−0.39	−0.37	−0.04	−0.76
	*p*		0.00 *	0.00 *	0.61	0.00 **
PDS	*r*			0.64	−0.12	0.39
	*p*			0.00 **	0.14	0.00 **
CDS	*r*				−0.14	0.39
	*p*				0.08	0.00 **
Drought	*r*					0.03
	*p*					0.73
K10	*r*					
	*p*					

** Significant at *p* ≤ 0.001, * Significant at *p* ≤ 0.05.

**Table 6:** Univariate analysis of sociodemographics with SOC13 and K10.

	SOC13 ^b^			K10		
Sociodemographics ^a^	Mean	SD	*p* (between Groups)	Mean	SD	*p* (between Groups)
**Gender**			0.11			0.48
Women	60.75	14.60		22.09	8.76	
Men	64.80	13.51		21.02	7.70	
**Age**			0.03 *			0.40
18–34	58.40	14.47		23.96	9.03	
35–54	59.55	13.96		21.74	8.04	
55+	65.36	14.65		21.25	8.87	
**Farmer Status**			0.70			0.93
Live and/or work on a farm	61.35	14.32		22.02	8.26	
Live in a rural community but not work or live on a farm	61.89	15.88		21.40	8.96	
Neither	66.50	11.93		22.17	12.40	
**Financial Position**			0.00 **			0.00 **
Prosperous/Comfortable	66.14	13.50		18.36	6.60	
Just getting along	59.59	14.50		23.79	8.79	
Poor/Very poor	51.47	12.47		28.47	8.51	
**Remoteness** (**Australian Standard Geographic Classification**)			0.07			0.13
Inner/Outer regional	63.26	13.62		21.10	8.20	
Remote/Very remote	59.04	15.69		23.18	8.95	

SD = Standard deviation. ^a^ Some categories are not consistent with Austin et al. [28] as they needed to be aggregated to account for a lack of power due to small sample sizes. ^b^ SOC13 strength: weak 13–63; and strong 64–91. ** Significant at *p* ≤ 0.001, * Significant at *p* ≤ 0.05.

**Table 7:** Linear regression showing the relationships between SOC13 and the influencing factors of sociodemographics, drought-related stress, drought and wellbeing.

	Model I ^a^		Model II ^a^		Model III ^a^	
	β	*p*	β	*p*	β	*p*
**Age**						
18–34	Reference group					
35–54	−0.01	0.90	−0.02	0.87	−0.07	0.37
55+	0.15	0.18	0.13	0.22	0.10	0.18
**Farmer Status**						
Live and/or work on a farm	Reference group					
Live in a rural community but not work or live on a farm	−0.07	0.40	−0.06	0.45	−0.04	0.51
Neither	0.00	0.98	0.02	0.78	0.05	0.34
**Financial Position**						
Poor/Very poor	Reference group					
Just getting along	0.26	0.04	0.24	0.06	0.08	0.37
Prosperous/Comfortable	0.41	0.00 **	0.40	0.00 **	0.06	0.51
**Remoteness** (**Australian Standard Geographic Classification**)						
Remote/Very remote	Reference group					
Inner/Outer regional	−0.05	0.48	−0.06	0.40	−0.04	0.44
**Drought-Related Stress**						
Personal (PDS)	−0.24	0.02 *	−0.24	0.02 *	−0.10	0.19
Community (CDS)	−0.14	0.17	−0.15	0.14	0.01	0.92
**Drought**						
Months below decile 1 (percent)			−0.10	0.20	−0.03	0.60
**Wellbeing**						
K10					−0.71	0.00 **

^a^ Adjusted R^2^: Model I 21.5%; Model II 21.8%; and Model III 59.3%, ** Significant at *p* ≤ 0.001, * Significant at *p* ≤ 0.05.

**Table 8:** Average SOC according to drought condition and level of psychological distress (K10) (note: weak SOC = 13–63; strong SOC = 64–91).

Psychological Distress	Drought Condition (Percent of Months below Decile 1 in the 24-Month Time Window)						
	0–5%	>5–10%	>10–15%	>15–20%	>20–25%	>25%	Total (Mean)
Low	-	-	77	75	75	-	76
Moderate	-	-	62	65	60	57	62
High	-	-	52	50	50	-	51
Total (mean)	-	-	62	62	61	57	

## References

[1] Kiem A.S., Austin E.K. (2013). Drought and the future of rural communities: Opportunities and challenges for climate change adaptation in regional Victoria, Australia. Glob. Environ. Chang..

[2] Folke C., Coldin J., Berkes F., Berkes F., Colding J., Folke C. (2002). Building resilience for adaptive capacity in social-ecological systems. Navigating Social-Ecological Systems: Building Resilience for Complexity and Change.

[3] Adger W.N., Brooks N., Bentham G., Agnew M., Eriksen S. (2004). New Indicators of Vulnerability and Adaptive Capacity. Final Project Report.

[4] Adger W.N., Vincent K. (2005). Uncertainty in adaptive capacity. C. R. Geosci..

[5] Adger W.N. (2006). Vulnerability. Glob. Environ. Chang..

[6] Gallopín G.C. (2006). Linkages between vulnerability, resilience, and adaptive capacity. Glob. Environ. Chang..

[7] Lockwood M., Raymond C.M., Oczkowski E., Morrison M. Measuring the dimensions of adaptive capacity: A psychometric approach. Ecol. Soc..

[8] Antonovsky A. (1979). Health, Stress and Coping.

[9] Pallant J.F., Lae L. (2002). Sense of coherence, well-being, coping and personality factors: Further evaluation of the sense of coherence scale. Pers. Individ. Differ..

[10] Griffiths C.A., Ryan P., Foster J.H. (2011). Thematic analysis of Antonovsky’s sense of coherence theory. Scand. J. Psychol..

[11] Eriksson M., Mittelmark M., Mittelmark M.B., Sagy S., Eriksson M., Bauer G.F., Pelikan J.M., Lindström B. (2017). The Sense of Coherence and its Measurement. The Handbook of Salutogenesis.

[12] Roy P., Tremblay G., Robertson S., Houle J. (2015). “Do it All by Myself”: A Salutogenic Approach of Masculine Health Practice Among Farming Men Coping With Stress. Am. J. Men Health.

[13] Antonovsky A. (1987). The salutogenic perspective: Toward a new view of health and illness. Advances.

[14] Cutter S.L., Barnes L., Berry M., Burton C., Evans E., Tate E. (2008). A place-based model for understanding community resilience to natural disasters. Glob. Environ. Chang..

[15] Vins H., Bell J., Saha S., Hess J.J. (2015). The Mental Health Outcomes of Drought: A Systematic Review and Causal Process Diagram. Int. J. Environ. Res. Public Health.

[16] Sartore G.-M., Kelly B., Stain H.J., Albrecht G., Higginbotham N. (2008). Control, uncertainty, and expectations for the future: A qualitative study of the impact of drought on a rural Australian community. Rural Remote Health.

[17] Antonovsky A. (1987). Unraveling the Mystery of Health: How People Manage Stress and Stay Well.

[18] Mittelmark M.B., Bauer G.F., Mittelmark M.B., Sagy S., Eriksson M., Bauer G.F., Pelikan J.M., Lindström B. (2017). The meanings of salutogenesis. The Handbook of Salutogenesis.

[19] Eriksson M., Lindström B. (2007). Antonovsky’s sense of coherence scale and its relation with quality of life: A systematic review. J. Epidemiol. Community Health.

[20] Eriksson M., Mittelmark M.B., Sagy S., Eriksson M., Bauer G.F., Pelikan J.M., Lindström B. (2017). The sense of coherence in the salutogenic model of health. The Handbook of Salutogenesis.

[21] Harris P.A., Taylor R., Thielke R., Payne J., Gonzalez N., Conde J.G. (2009). Research electronic data capture (REDCap)—A metadata-driven methodology and workflow process for providing translational research informatics support. J. Biomed. Inform..

[22] CRRMH (2019). Centre for Rural and Remote Mental Health: Centre for Rural and Remote Mental Health (CRRMH). https://www.crrmh.com.au/.

[23] CRRMH (2019). Rural Adversity Mental Health Program (RAMHP): Centre for Rural and Remote Mental Health (CRRMH). https://www.crrmh.com.au/programs-and-projects/ramhp/.

[24] UON (2018). University Communications and Media: University of Newcastle (UON). https://www.newcastle.edu.au/our-uni/contact/media-centre#news-events.

[25] Snider J.G., Osgood C.E. (1969). Semantic Differential Technique: A Sourcebook.

[26] DePoy E., Gitlin L.N., DePoy E., Gitlin L.N. (2016). Chapter 17—Collecting Data Through Measurement in Experimental-Type Research. Introduction to Research.

[27] Jaakkola S., Rautava P., Saarinen M., Lahti S., Mattila M.-L., Suominen S. (2013). Dental fear and sense of coherence among 18-yr-old adolescents in Finland. Eur. J. Oral. Sci..

[28] Austin E.K., Handley T., Kiem A.S., Rich J.L., Lewin T.J., Askland H.H. (2018). Drought-related stress among farmers: Findings from the Australian Rural Mental Health Study. Med. J. Aust..

[29] Austin E.K., Kiem A.S., Rich J.L., Perkins D., Kelly B.J. (2020). How Effectively Do Drought Indices Capture Health Outcomes? An Investigation from Rural Australia. Review.

[30] Jones D., Wang W., Fawcett R. (2009). High-quality spatial climate data-sets for Australia. Aust. Meteorol. Oceanogr. J..

[31] Tozer C.R., Kiem A.S., Verdon-Kidd D.C. (2012). On the uncertainties associated with using gridded rainfall data as a proxy for observed. Hydrol. Earth Syst. Sci..

[32] Schober P., Boer C., Schwarte L.A. (2018). Correlation Coefficients: Appropriate Use and Interpretation. Anesth. Analg..

[33] ABS (2018). 1270.0.55.005—Australian Statistical Geography Standard (ASGS): Volume 5—Remoteness Structure, July 2016: Australian Bureau of Statistics (ABS). https://www.abs.gov.au/AUSSTATS/abs@.nsf/DetailsPage/1270.0.55.005July%202016?OpenDocument.

[34] ABS 4364.0.55.001—National Health Survey: First Results, 2017–2018—Australian Bureau of Statistics (ABS); 2018 [updated 12.12.2018]. https://www.abs.gov.au/ausstats/abs@.nsf/Lookup/by%20Subject/4364.0.55.001~2017-18~Main%20Features~Psychological%20distress~20.

[35] Austin E.K., Rich J.L., Kiem A.S., Handley T.E., Perkins D., Kelly B. (2020). Concerns about climate change among rural residents in Australia. J. Rural. Stud..

[36] Eriksson M., Lindström B., Lilja J. (2007). A sense of coherence and health. Salutogenesis in a societal context: Aland, a special case?. J. Epidemiol. Community Health.

[37] Tselebis A., Moulou A., Ilias I. (2001). Burnout versus depression and sense of coherence: Study of Greek nursing staff. Nurs. Health Sci..

[38] Cederfjäll C., Langius-Eklöf A., Lidman K., Wredling R. (2001). Gender Differences in Perceived Health-Related Quality of Life Among Patients with HIV Infection. AIDS Patient Care STDS.

[39] Sanan A., Quinn C., Spiegel J.H. (2013). Patient Preferences in Print Advertisement Marketing for Plastic Surgery. Aesthetic Surg. J..

[40] Rich J.L., Handley T.E., Inder K., Perkins D. (2018). An experiment in using open-text comments from the Australian Rural Mental Health Study on health service priorities. Rural Remote Health.

[41] Foote C. (2009). It’s a Mad, Mad Wordle: For a New Take on Text, Try This Fun Word Cloud Generator. Sch. Libr. J..

[42] Motzer S.A., Hertig V., Jarrett M., Heitkemper M.M. (2003). Sense of Coherence and Quality of Life in Women With and Without Irritable Bowel Syndrome. Nurs. Res..

[43] Stain H.J., Kelly B., Carr V.J., Lewin T.J., Fitzgerald M., Fragar L. (2011). The psychological impact of chronic environmental adversity: Responding to prolonged drought. Soc. Sci. Med..

[44] MacLeod A.K., Byrne A. (1996). Anxiety, depression, and the anticipation of future positive and negative experiences. J. Abnorm. Psychol..

[45] Ellis N., Albrecht G.A. (2017). Climate change threats to family farmers’ sense of place and mental wellbeing: A case study from the Western Australian Wheatbelt. Soc. Sci. Med..

[46] BCG Critical Breaking Point? The Effects of Drought and Other Pressures on Farming Families. Birchip Cropping Group (BCG). www.bcg.org.au/cb_pages/SocialResearchProjects.php.

